# Prevalence, predictors and clinical significance of *Blastocystis* sp. in Sebha, Libya

**DOI:** 10.1186/1756-3305-6-86

**Published:** 2013-04-08

**Authors:** Awatif M Abdulsalam, Init Ithoi, Hesham M Al-Mekhlafi, Abdul Hafeez Khan, Abdulhamid Ahmed, Johari Surin, Joon Wah Mak

**Affiliations:** 1 Department of Parasitology, Faculty of Medicine, University of Malaya, Kuala Lumpur 50603, Malaysia; 2 Department of Parasitology, Faculty of Medicine, University of Sebha, Sebha, Libya; 3 Department of Biology, Faculty of Natural and Applied Sciences, Umaru Musa Yar’adua University, Katsina, Katsina State, Nigeria; 4 School of Postgraduate Studies and Research, International Medical University, Bukit Jalil, Kuala Lumpur, 57000, Malaysia; 5 Department of Parasitology, Faculty of Medicine and Health Sciences, Sana’a University, Sana’a, Yemen

**Keywords:** *Blastocystis*, Gastrointestinal symptoms, Sebha, Libya

## Abstract

**Background:**

*Blastocystis* sp. has a worldwide distribution and is often the most common human intestinal protozoan reported in children and adults in developing countries. The clinical relevance of *Blastocystis* sp. remains controversial. This study was undertaken to determine the prevalence of *Blastocystis* infection and its association with gastrointestinal symptoms among outpatients in Sebha city, Libya.

**Methods:**

A total of 380 stool samples were collected from outpatients attending the Central Laboratory in Sebha, Libya for routine stool examination. The presence of *Blastocystis* sp. was screened comparing light microscopy of direct smears against in vitro cultivation. Demographic and socioeconomic information were collected with a standardized questionnaire.

**Results:**

The overall prevalence of *Blastocystis* infection was 22.1%. The prevalence was significantly higher among patients aged ≥18 years compared to those aged < 18 years (29.4% vs 9.9%; *x*^*2*^ = 19.746; *P* < 0.001), and in males compared to females (26.4% vs 17.5%; *x*^*2*^ = 4.374; *P* = 0.036). Univariate analysis showed significant associations between *Blastocystis* infection and the occupational status (*P* = 0.017), family size (*P* = 0.023) and educational level (*P* = 0.042) of the participants. Multiple logistic regression analysis confirmed that the age of ≥ 18 years (OR = 5.7; 95% CI = 2.21; 9.86) and occupational status (OR = 2.2; 95% CI = 1.02, 4.70) as significant predictors of *Blastocystis* infection among this population. In those who had only *Blastocystis* infection but no other gastrointestinal parasitic infections, the prevalence of gastrointestinal symptoms was higher compared to those without *Blastocystis* infection (35.3% vs 13.2%; *x*^*2*^ = 25.8; *P <* 0.001). The most common symptoms among these patients were abdominal pain (76.4%), flatulence (41.1%) and diarrhoea (21.5%).

**Conclusions:**

*Blastocystis* sp. is prevalent and associated with gastrointestinal symptoms among communities in Sebha city, Libya. Age and occupational status were the significant predictors of infection. However, more studies from different areas in Libya are needed in order to delineate the epidemiology and clinical significance of this infection.

## Background

*Blastocystis* sp. is one of the most common intestinal protozoa found in the human intestinal tract. *Blastocystis* infection is widely distributed throughout the world with a high prevalence in developing countries in the tropics and subtropics [1,2]. Human infection is associated with poor personal hygiene, lack of sanitation, exposure to animals, and consumption of contaminated food or water [3,4]. Although it was discovered almost a century ago *Blastocystis* pathogenicity is reported to be controversial [4-6]. The clinical manifestations among the symptomatic individuals are mainly nonspecific such as diarrhoea, abdominal pain, nausea, fatigue, vomiting, anorexia, and flatulence [3,4,6]. The routine diagnosis of the infection is currently based on microscopic identification of the protozoa in the direct smears carried out before or after cultivation of the faecal sample [4].

In Libya, previous studies in Sebha, city showed that *Blastocystis* sp. was frequently isolated from symptomatic patients [7,8]. Detection of the *Blastocystis* sp. is not routinely performed in most Libyan laboratories, so the prevalence and predictors of *Blastocystis* infection are poorly known. Given the lack of epidemiological information on *Blastocystis* sp. in Libya, this study was undertaken to determine the prevalence and associated predictors of *Blastocystis* infection among outpatients attending the Central Laboratory in Sebha, Libya.

## Methods

### Study area and study population

**Figure 1:**
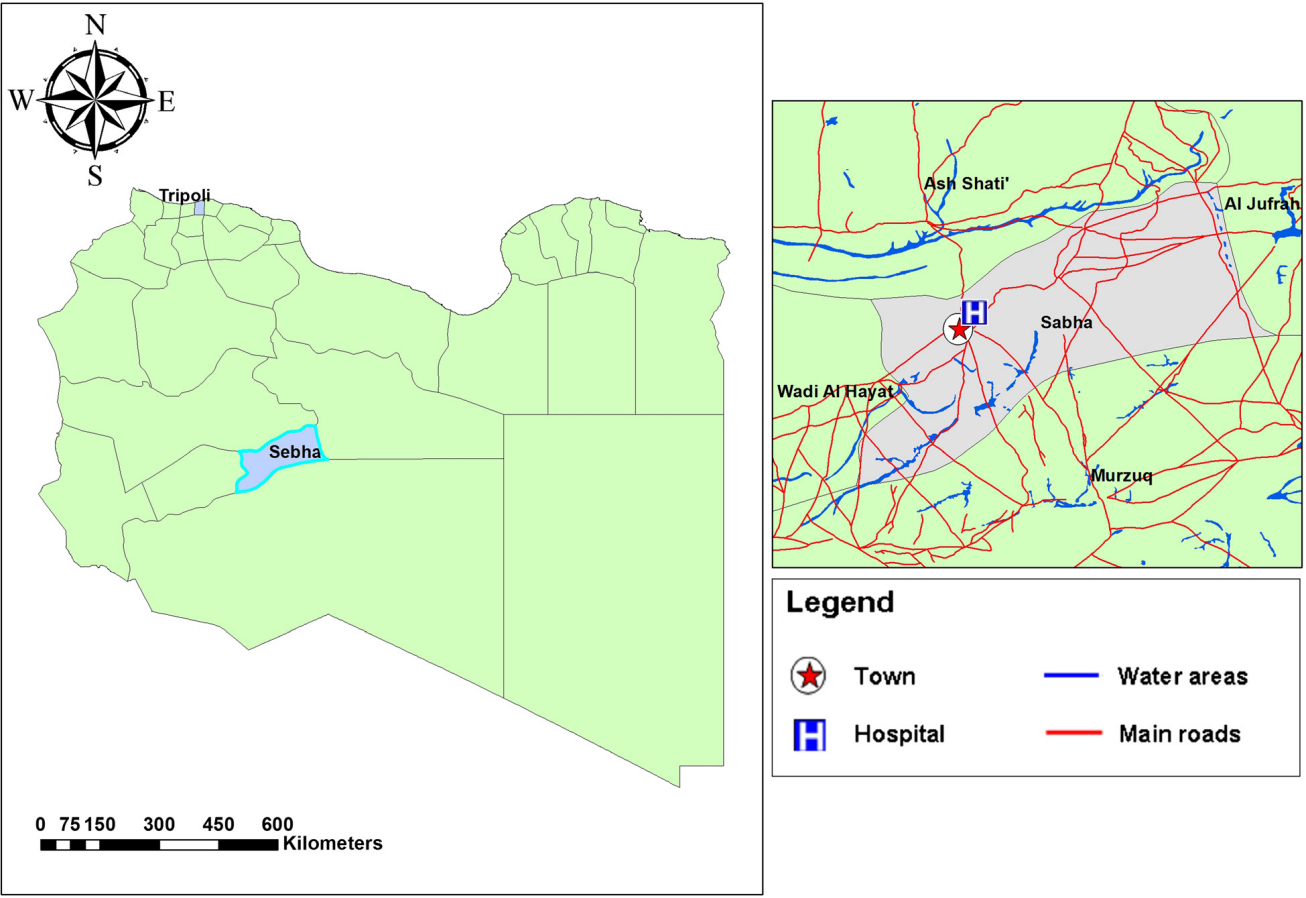
A geographic map showing Libya and the location of Sebha city.

This cross-sectional study was carried out in Sebha city, Libya, about 800 km south of Tripoli (longitude 14.42^o^E, latitude 27.03^o^N) (Figure 1). The city is situated in the Fezzan valley with a total area of 15,330 km^2^ and a total population of 130,000 people. The area is characterized by desert climate, dry and hot weather and low rainfall. Agriculture is the main occupation of the people and underground wells are the main source of water. Data collection was carried out between August and November 2010.

A total of 380 stool samples were collected from outpatients at Sebha Central Laboratory. The samples were collected as a part of a routine medical examination of people living in the city or within the vicinity. Prior to data collection, the nature of the study was explained to the participants and informed verbal consents were obtained. Demographic, socioeconomic, environmental and behavioural information and history of gastrointestinal (GI) symptoms were collected with a standardized questionnaire (face-to-face interviews). Participants who tested positive for *Blastocystis* infection were divided into symptomatic hosts (n = 54) or asymptomatic hosts (n = 30), based on the presence or absence of GI symptoms. The protocol of this study was approved by the Medical Ethics Committee of the University of Malaya Medical Center, Kuala Lumpur. Based on this ethical clearance, permission to conduct this study was also given by the Faculty of Medicine, University of Sebha and Sebha Central laboratory authorities before the commencement of the study.

### Stool examination

A single faecal sample was collected from each patient in a clean plastic container. The specimens were examined for the presence of intestinal parasites and cultured for *Blastocystis* sp. at the Central Laboratory, Sebha, Libya. No further information was available on potential viral or bacterial infections.

### Detection of *Blastocystis* sp

The fecal samples were cultured in Jones’ medium supplemented with 10% horse serum [9]. For each culture, approximately 50 mg of stool was inoculated into a 15-ml screw-cap tube containing 5 ml of Jones’ medium. All inoculated tubes were tightly-closed, placed in a rack and incubated at 37°C. The medium in each of these tubes was replaced with the new complete Jones’ medium every alternate day starting from day 2 of cultivation. This was carried out by discarding about 4.0 ml of the medium at the top level (without disturbing the pellet) and replaced by 4.0 ml of new complete Jones’ medium. The presence of *Blastocystis* sp. was observed daily for 14 days of cultivation, by placing 1 drop of the cultured sediment onto a glass slide, covered with a cover-slip and viewed (X100 and X400 objectives) under light microscopy. Positive cultures were defined by the detection of any form of *Blastocystis* sp. (i.e. vacuolar, granular, amoeboid, and cystic forms), as in our previous published report [3].

### Detection of other intestinal parasites

The stool samples were examined on the day of collection. Wet mount preparations of the stool samples were examined using light microscopy. In addition, specimens were concentrated by a formalin-ethyl acetate sedimentation technique. Sediments were then examined as a wet mount in saline and iodine for detection of protozoa, eggs and larvae of intestinal helminthes. Permanent stained smears were carried out by the modified Ziehl-Neelsen stain for intestinal coccidian parasites [10].

### Statistical analysis

Statistical analysis was performed using the Statistical Package for Social Sciences for Windows (SPSS), version 11.5 (SPSS Inc, Chicago, IL, USA). Demographic and socioeconomic characteristics were treated as categorical variables and presented as frequencies and percentages. Pearson’s Chi Square test was used to examine the associations of *Blastocystis* prevalence with the demographic and socioeconomic factors. Odds ratio (OR) and 95% confidence intervals (CI) were computed. A multiple logistic regression model was performed to identify the significant predictors of infection. All P values ≤ 0.05 were considered statistically significant.

## Results

In this study, stool specimens were collected from a total of 380 patients (197 males and 183 females) aged from 1 to 75 years (median age = 25 years, inter-quartile range = 9–41 years). The general characteristics of the patients are shown in Table 1. The patients were from Sebha city and its surroundings. Almost half of the participants had a low level of education (31.6% had no formal education and 17.6% had primary education). Similarly, about half of them had high level of education (39.5% had secondary education and 11.3% had university degree). Moreover, all of the houses are built of bricks and concrete and had electricity, piped water and a flush toilet facility. More than half of patient enrolled in the study were asymptomatic while the rest were symptomatic with one or more of following gastrointestinal symptoms: abdominal pain, diarrhoea, flatulence, constipation, vomiting, and nausea.

**Table 1:** General characteristics of the participants (n = 380)

**Characteristics**	**Frequency (%)**
**Age group (years):**	
≥18	238 (62.6)
<18	142 (37.4)
**Gender:**	
Male	197 (51.8)
Female	183 (48.2)
**Socioeconomic status:**	
Participants’ education level (at least 6 years)	120 (31.6)
Working participants	132 (44.0)
Large family size (≥ 7 members)	222 (58.4)
Treated drinking water (filtered or boiled)	138 (36.3)
Presence of animals in the house	44 (11.6)
History of recent overseas travel	16 (4.2)
Presence of gastrointestinal (GI) symptoms	153 (40.3)

### Prevalence and predictors of *Blastocystis* infection

The overall prevalence of *Blastocystis* infection was 22.1% (84/380). The association of *Blastocystis* infection with the demographic and socioeconomic factors was examined using univariate analysis and the results are presented in Table 2. The results showed that the participants aged ≥ 18 years had a higher prevalence of *Blastocystis* infection than those aged < 18 years (29.4% vs 9.9%; x^2^ = 19.746; *P* < 0.001). Similarly, a significantly higher prevalence of infection was reported in males as compared to females (26.4% vs 17.5%; *x*^*2*^ = 4.374; *P* = 0.036). The prevalence of infection was significantly higher among those who were working (31.8% vs 19.6%; *x*^*2*^ = 5.848; *P* = 0.017) and had low educational level (26.4% vs 17.6%; *x*^*2*^ = 4.250; *P* = 0.042) compared to their counterparts. On the other hand, those living in families with ≥ 7 members had significantly lower prevalence of *Blastocystis* infection than those living in small families (18.0% vs 27.8; *x*^*2*^ = 5.180; *P* = 0.023). Multiple logistic regression analysis confirmed the age of ≥ 18 years (OR = 5.7; 95% CI = 2.21, 9.86) and working status (OR = 2.2; 95% CI = 1.02, 4.70) as significant predictors of blastocystosis among this population (Table 3). Other variables including type of drinking water, presence of animals at the household and travel history showed no significant association with the prevalence of *Blastocystis* infection.

**Table 2:** **Univariate analysis of potential predictors for *Blastocystis *infection among the participants (n = 380)**

**Variable**	***Blastocystis *infection**		**OR (95% CI)**	***P***
	**No. Examined**	**% Infected**		
**Age**				
≥18 years	238	29.4	3.8 (2.05, 7.06)	<0.001*
<18 years	142	9.9	1	
**Gender**				
Male	197	26.4	1.7 (1.03, 2.77)	0.036 *
Female	183	17.5	1	
**Education level**				
≤ Primary school	193	26.4	1.7 (1.02, 2.75)	0.042*
≥ Secondary school	187	17.6	1	
**Occupational status**				
Working	132	31.8	2.0 (1.13, 3.24)	0.017*
Not working	168	19.6	1	
**Family size**				
≥ 7 members (large)	222	18.0	0.6 (0.35, 0.93)	0.023*
< 7 members	158	27.8	1	
**Drinking water**				
Untreated water	242	23.1	1.2 (0.70, 1.97)	0.520
Treated water (chemical, filtered or boiled)	138	20.3	1	
**Presence of animals** **in the house**				
Yes	44	13.6	0.5 (0.21, 1.28)	0.150
No	336	23.3	1	
**History of recent overseas travel**				
Yes	16	31.3	1.6 (0.55, 4.85)	0.360
No	364	21.7	1	
**Presence of GI symptoms**				
Yes	153	35.3	3.6 (2.14, 5.94)	<0.001*
No	227	13.2	1	
**Abdominal pain**				
Yes	131	35.9	3.2 (1.94, 5.28)	<0.001*
No	249	14.9	1	
**Diarrhoea**				
Yes	41	31.7	1.8 (0.86, 3.56)	0.117
No	339	20.9	1	
**Flatulence**				
Yes	55	49.1	4.5 (2.48, 8.26)	<0.001*
No	325	17.5	1	

*OR*, odds ratio; *CI*, confidence interval.

* Significant association (*P* < 0.05).

**Table 3:** **Results of multivariate analysis of potential predictors for *Blastocystis *infection among the participants (n = 380)**

**Variables**	***Blastocystis *infection**	***P***
	**Adjusted OR (95% CI)**	
Age (≥ 18 years)	5.7 (2.21, 9.86)	0.001*
Gender (male)	1.6 (0.67, 3.55)	0.304
Educational level (≤ primary education)	1.8 (0.86, 4.26)	0.113
Occupational status (working)	2.2 (1.02, 4.70)	0.045*
Family size (small)	0.7 (0.32, 1.07)	0.063

*OR*, odds ratio; *CI*, confidence interval.

^* ^Significant predictors (*P* ≤ 0.05).

### Single and multiple infections

Of 380 patients examined, 91 (24%) were positive for intestinal protozoa. Of these 91 patients, 79 patients were singly infected with *Blastocystis* sp., 5 patients were infected with *Blastocystis* sp. concurrently with four species of intestinal parasites namely *Giardia duodenalis, Entamoeba histolytica/dispar, Crptosporidium* spp. and *Enterobius vermicularis*. Moreover, 7 patients were *Blastocystis*-negative but infected with either *Giardia duodenalis* or *Entamoeba histolytica/dispar*.

### Symptoms

Most gastrointestinal symptoms reported by symptomatic patients were nonspecific and included diarrhoea, abdominal pain, flatulence, constipation, nausea, and vomiting. The prevalence of *Blastocystis* infection was significantly higher among the symptomatic subjects compared to the asymptomatic subjects (35.3% vs 13.2%; *x*^*2*^ = 25.874; *P* < 0.001). In the group examined, 64.3% (54/84) of the *Blastocystis*-positive patients were symptomatic and 35.7% (30/84) were asymptomatic. *Blastocystis* sp. represented the only intestinal parasite in 51 of these symptomatic patients. The common symptoms among patients infected exclusively with *Blastocystis* sp. were abdominal pain (76.4%), flatulence (41.1%) and diarrhoea (21.5%). Moreover, 5.8% and 3.9% of these patients had nausea/vomiting and constipation, respectively. Twenty one of the symptomatic patients (41.2%) had two or more GI symptoms while 35.3% had only one symptom. Abdominal pain (35.9% vs 14.9%; *x*^*2*^ = 22.023; *P* < 0.001) and flatulence (49.1% vs 17.5%; *x*^*2*^ = 27.197; *P* < 0.001) were the significant symptoms associated with *Blastocystis* infection observed in the subjects studied (Table 2).

## Discussion

In the present study, the prevalence of *Blastocystis* infection was 22.1%, which is within the range of the prevalence rate of previous studies in Libya [7,8,11,12]. The prevalence of *Blastocystis* infection in Sebha was reported to range between 18.5% and 26.2% [7,8]. Moreover, studies from other regions of Libya have reported a prevalence of 29.6% in Sirt city [11] and 6.7% among schoolchildren in Derna [12]. Most Libyan medical practitioners are not familiar with *Blastocystis* infection in humans, even though *Blastocystis* infection was commonly found in hospitalized patients. Moreover, knowledge on the epidemiology and transmission of *Blastocystis* sp. is not widely known and thus the laboratory detection of *Blastocystis* sp. in stool samples is not routinely carried out.

Shedding of *Blastocystis* sp. from infected individuals especially asymptomatic carriers could be a source of infection in the region. An enormous increase of foreign workers from neighbouring countries (mainly Egypt, Sudan and Chad) and travellers may have contributed to the high prevalence of intestinal parasites especially *Blastocystis* sp. in the country. However, the prevalence of intestinal parasitic infections is expected to increase due to the civil unrest and war in Libya since early 2011. Although there were other intestinal protozoa parasites found in these participants, *Blastocystis* sp. was the most common. Other intestinal protozoa found included *Giardia duodenalis*, *Cryptosporidium spp.*, *Entamoeba histolytica, Entamoeba coli* (not reported in the results). Moreover, *Enterobius vermicularis* was the only helminth detected in one patient. It seems that the arid climate of the study area is not favourable for the development of helminth parasites which need either moist soil (such as soil-transmitted helminths) or aquatic environment where intermediate hosts such as snails, fish or aquatic plants can be found (such as *Schistosoma spp.*, Liver flukes, *Diphyllobothrium latum*, …etc.) [13].

The present study is the first to provide information about the predictors of *Blastocystis* infection in Libya. Previous reports from different countries have shown that *Blastocystis* infections are associated with several factors such as the consumption of contaminated food and water, close contact with animals, poor personal hygiene, inadequate sanitation, geographical distribution, agricultural activities and seasonal influences [3,4,14-16]. Our findings showed that adult participants (aged ≥18 years) were almost 4 times more likely to be infected with *Blastocystis* sp. It was also found that males were more prone to be infected than females. Several studies have also reported a significantly higher prevalence in male than female patients in Libya [8,17] and other countries [5,18]. Outdoor activities by the adult males may also explain the significantly higher prevalence of *Blastocystis* infections among these groups. Previous studies have found significantly higher infection rates in adults than in children with the highest prevalence rate among young adults aged between 18 and 30 years [1,19,20]. In contrast, other reports found a higher prevalence rate in children and females as compared to adults and males [21-23]. Moreover, a recent study has reported a significant reduction in the *Blastocystis* infection prevalence rate in older children when compared with younger children [9]. These contradictory findings suggest that the distribution of *Blastocystis* infection shows spatial heterogeneity with respect to age or sex factors. The age and gender correlations identified in this study may not represent physiological properties intrinsic to those hosts, but rather may be caused by the variation in environmental conditions associated with age and gender.

In the same vein, the present study showed that occupational status of the participants was a significant predictor of *Blastocystis* infection, with those employed having a greater odds for infection compared to those unemployed. The high prevalence rate among employed participants may indicate a higher exposure to the source of infection at the work places including the food and environment. Supporting this conjecture, a high prevalence rate of *Blastocystis* infection (35.5%) was reported among food handlers in Libya [24].

Our findings showed a significant association between the educational level of the participants and the prevalence of *Blastocystis* infection. Improvement of hygienic conditions and sanitary practices with education is well documented and several previous studies have identified that the low level of education as a significant risk factor of blastocytosis and other parasitic infections [3,25,26]. Another significant finding of the present study that was associated with the risk of *Blastocystis* infection was the family size. Participants belong to large families (≥7 members) were at lower odds for *Blastocystis* infection compared to those from smaller families. This finding is contrary to previous studies that reported a significant association between *Blastocystis* infection and the presence of other infected family members [27] and this has been attributed to the horizontal spread or the focal transmission of infection among family members in the vicinity of the home. Nevertheless, these significant associations of *Blastocystis* infection with the level of education and family size were not confirmed by the multiple logistic regression analysis.

*Blastocystis* infection was noted to be transmitted through the faecal-oral route [28], contaminated water [3,27], and food [29,30]. The environmentally resistant cyst represents the transmissible form of the parasite [4,31,32]. Drinking contaminated water, especially surface water, was reported to be a significant risk factor for *Blastocystis* infection [3,14,27,33]. The current study found that all houses had a piped water supply (groundwater) and almost two-thirds of the participants use this water for drinking and other domestic household purposes such as cooking without any physical or chemical treatment. However, we found no significant difference in the prevalence of *Blastocystis* infection between those who use treated water and those who use untreated water. This may indicate that the level of *Blastocystis* contamination in groundwater is low. Data about the presence of waterborne parasites in drinking water and sewage in Sebha city are not available. Similarly, the present study found no significant association between *Blastocystis* infection and contact with animals. A significantly higher prevalence of *Blastocystis* infection among people having close contact with animals was reported by previous studies [15,16]. Molecular studies have also showed that *Blastocystis* isolates from animals are found in the human host [15,34,35]. Hence, the possibility of waterborne zoonotic transmission of *Blastocystis* sp. was highly suggested [16].

Sebha city has adequate public services and other infrastructure with the exception of health structures. It was observed that the garbage and sewage from the city are discharged without proper treatment in the nearby desert land and this may have an influence on the contamination of the environment with cysts of *Blastocystis* sp. and other enteric pathogenic parasites (e.g. *Cryptosporidium, Giardia*). Moreover, this unhygienic practice may facilitate the transfer of the infective stages through wind into the food and environment. Besides, *Blastocystis* infection was reported to be most common during summer [8], where, the hot and dry climate may increase the transmission of the resistant forms (cyst or oocyst) of enteric protozoa (especially *Blastocystis* sp.) in the dusty environments of this city. This may be also partially explain the higher prevalence in males, who work more outdoors and therefore, have more exposure to the source of infections compared to females.

Numerous studies have examined the pathogenic potential of *Blastocystis* sp. by investigation its prevalence in symptomatic and asymptomatic groups, and have thus either supported or denied pathogenic significance of this protozoan [21,36-38]. In the present study, we found a strong statistical association between the infection and the development of gastrointestinal symptoms (*P* < 0.001). The most common symptoms in symptomatic patients were abdominal pain 76.4%, flatulence 41.1%, and diarrhoea 21.5%. These findings are consistent with previous studies [5,38,39]. In Saudi Arabia, abdominal pain (87.9%), constipation (32.2%) and diarrhoea (23.4%) were reported among 12,136 *Blastocystis*-infected patients [20]. Moreover, abdominal pain (76.9%), diarrhoea (50%) and distention (32.6%) were found to be associated with *Blastocystis* infection among hospitalized children in Turkey [39]. Similarly, abdominal pain, recurrent diarrhoea, cramps, anorexia, and fatigue were significantly associated with *Blastocystis* infection among preschool children in Jordan [5]. Moreover, the majority of the patients in our study had two gastrointestinal symptoms, which is consistent with several reports among patients infected with *Blastocystis* sp. [6,18]. However, a previous report on the *Blastocystis* associated symptoms among patients from Sebha city found a higher number of diarrhoea cases followed by abdominal pain, flatulence and nausea or vomiting [8].

It has been suggested that *Blastocystis sp.* may be an opportunistic pathogen in immunocompromised individuals including AIDS patients and individuals with cancer [40,41]. However, the correlation between clinical symptoms and *Blastocystis* in immunocompromised individuals could not be delineated and the possibility of other unidentified etiological agents, especially viruses, toxins, and non infectious causes could not be assessed. As a limitation of the present study, data on the immunological status of the participants were not available. The study may have incorporated a selection bias, as only samples from patients submitted to the Sebha Central Laboratory were examined. This limitation is difficult to overcome, as collecting faecal samples from the general population of this Libyan community has been found to be difficult. However, Sebha Central Laboratory receives samples from symptomatic and asymptomatic individuals including those who come for routine medical check ups requested by public and private organizations and educational institutions. Thus, we may speculate that the findings of the present study can be generalized to the Sebha population. On the other hand, these findings cannot be generalized to the entire Libyan population. However, further studies are required to confirm these conjectures.

## Conclusions

This study reveals a high prevalence of *Blastocystis* infection among individuals seeking health care in Sebha city in Libya. The age of ≥18 years and occupational status were the significant predictors of infection in this population. A significant association between symptoms and *Blastocystis* infections was reported. Detection of *Blastocystis* sp. is not routinely performed in most Libyan laboratories; hence, laboratory technicians need to be trained in the detection of *Blastocystis* sp. in clinical samples. Further studies on animal and environmental isolates are required to identify different transmission routes and reservoirs of *Blastocystis* sp. In addition, more research especially those that utilize advanced molecular techniques are highly recommended in future attempts to reveal the clinical significance of the different *Blastocystis* sp. subtypes.

## Competing interests

The authors declare that they have no competing interests.

## Authors’ contributions

AMA was involved in all phases of the study, including data collection and analysis, interpretation, and write up of the manuscript; II and HMA designed and supervised the study and; AMA, AHK, AA were involved in the collection and laboratory examination of samples. MJW and JS revised the analysis and manuscript. All authors read and approved the final manuscript.
